# Welcome to *Rheumatology Advances in Practice*

**DOI:** 10.1093/rap/rkx001

**Published:** 2017-06-05

**Authors:** Richard A. Watts

**Affiliations:** Editor-in-Chief Consultant Rheumatologist, Ipswich Hospital NHS Trust Honorary Clinical Senior Lecturer, University of East Anglia.

*Rheumatology Advances in Practice* is a new online open access journal published by the British Society for Rheumatology (BSR) and Oxford University Press. The journal will complement the well-established journal *Rheumatology* and reflect the increasing breadth of rheumatology practice. In many countries a growing diversity of non-medical professionals including specialist nurses, physiotherapists, podiatrists and pharmacists play an important role in the management of patients with rheumatic diseases. The close relationship between these disciplines has recently been emphasised in the UK by the merger of professional bodies representing allied health professionals and primary care physicians into the British Society for Rheumatology. The BSR has therefore decided that to better reflect its broader membership, it will support a second journal.

With the expansion of non-medical care in rheumatic diseases has come an increase in academic and clinical research by the various related disciplines. The aim of *Rheumatology Advances in Practice* is to reflect this growing research activity and to provide a truly multidisciplinary journal for this community, which we consider is not adequately served by existing publications. The core editorial team therefore has members with a physiotherapy, psychology and nursing backgrounds. Further details of the team can be found at the journal website (https://academic.oup.com/rheumap).

Our key aim is to provide rapid publication of high quality papers covering therapies, imaging, education, qualitative research, health economics, rare rheumatic diseases, and translational science, and we very much welcome papers from all healthcare professionals and BSR members. Published manuscripts from non-medical health care professionals will attract a lower open access article publication charge. As soon as papers have been fully peer reviewed and accepted, they will be published online, prior to copy-editing and typesetting, to facilitate the dissemination of papers rapidly. The final version of the paper will be subsequently posted on line following copyediting, typesetting and correction by the authors. Peer review will be rigorous and to the same high standard as *Rheumatology* using single blind review. We believe that this combination will enable us to achieve rapid publication of high quality papers.

On behalf of the editorial team, the BSR and Oxford University Press we look forward to receiving your manuscripts and to publishing them in *Rheumatology Advances in Practice*.

